# Effectiveness of Hypnosis in Combination with Conventional Techniques of Behavior Management in Anxiety/Pain Reduction during Dental Anesthetic Infiltration

**DOI:** 10.1155/2017/1434015

**Published:** 2017-04-11

**Authors:** A. Ramírez-Carrasco, C. Butrón-Téllez Girón, O. Sanchez-Armass, M. Pierdant-Pérez

**Affiliations:** ^1^Posgrado de Estomatología Pediátrica, Facultad de Estomatología, Universidad Autónoma de San Luis Potosí, Avenida Manuel Nava 2, 78290 San Luis Potosí, SLP, Mexico; ^2^Laboratorio de Bioquímica, Microbiología y Patología, Facultad de Estomatología, Universidad Autónoma de San Luis Potosí, Avenida Manuel Nava 2, 78290 San Luis Potosí, SLP, Mexico; ^3^Facultad de Psicología, Universidad Autónoma de San Luis Potosí, De Los Talleres 186, Valle Dorado, 78399 San Luis Potosí, SLP, Mexico; ^4^Departamento de Epidemiología Clínica, Facultad de Medicina, Universidad Autónoma de San Luis Potosí, Avenida Venustiano Carranza 2405, 78210 San Luis Potosí, SLP, Mexico

## Abstract

*Background and Objective*. Anxiety/pain are experiences that make dental treatment difficult for children, especially during the time of anesthesia. Hypnosis is used in pediatric clinical situations to modify thinking, behavior, and perception as well as, recently, in dentistry; therefore the aim of this study was to evaluate the effectiveness of hypnosis combined with conventional behavior management techniques during infiltration anesthetic.* Methods*. Anxiety/pain were assessed with the FLACC scale during the anesthetic moment, as well as heart rate variability and skin conductance before and during the anesthetic moment, between the control and experimental group.* Results*. A marginal statistical difference (*p* = 0.05) was found in the heart rate between baseline and anesthetic moment, being lower in the hypnosis group. No statistically significant differences were found with the FLACC scale or in the skin conductance (*p* > 0.05). *Conclusion*. Hypnosis combined with conventional behavior management techniques decreases heart rate during anesthetic infiltration showing that there may be an improvement in anxiety/pain control through hypnotic therapy.

## 1. Introduction

Fear and anxiety are the principal obstacles for dental treatment in children and can turn into dental phobia, leading to patients avoiding dental treatment [1]. While dentistry has benefitted from technological advances, the anxiety related to the environment in a dentist's office and, specifically, to the dental treatment of children is a global problem and, thus, poses a significant challenge for the provision of dental care [2]. Thus, the anxiety related to dental treatment and fear-related behaviors are some of the most difficult aspects for the management of a child patient [3, 4]. The prevalence of dental anxiety in children and adolescents varies extensively from 5 to 40% [2] and tends to decrease with age [2]. The relationship between anxiety and pain is of the utmost importance in that, for pain to develop, a physiological component and an intense cognitive factor are necessary. Those children who experience anxiety when faced with dental treatment generally have exaggerated experiences and perceptions of pain [5]. Pain is a difficult experience to evaluate objectively, in that it is a combination of unpleasant sensations and emotions, which, in a child, vary according to cognitive, emotional, and social experiences [6]. Dentistry involves numerous procedures which can be perceived as painful for a child, with the administration of anesthetics and extractions being the most painful of all, potentially causing psychological distress [7]. The FLACC (Face, Legs, Activity, Cry, Consolability) scale for the evaluation of pain demonstrates a good level of reliability and validity both during and after the surgical and medical procedures and trauma and malignant processes that produce pain in children, both in small and older children [8, 9]. Behavioral control is an essential part of the management of children in the dentist's office, on which the cooperation of the child and, thus, the quality of the dental treatments undertaken depend [10], with the main objective of behavioral management being the relief of fear and anxiety [11].

Hypnosis is widely and often successfully used in a variety of clinical pediatric situations to modify patients' thinking, behavior, and perception [12, 13]. It is one of the options being used in dentistry as a method to help the anxious subject relax [14] and could be used to improve the level of patient cooperation by increasing confidence itself [12]. Recently, dentistry is one of the medical fields more accepting of hypnosis, as it has been very effective in controlling “toothache”; furthermore it is being used in oral surgery as a complement to anesthesia, with its main tools being suggestion and speech [15]. For this reason, hypnosis is an option for reducing the anxiety or pain associated with infiltration anesthesia and, together with the standard behavior management techniques used in pediatric dentistry, could be useful for the management of pediatric patient behavior during dental treatment.

The purpose of this study was to evaluate the efficacy of hypnosis used with behavior management techniques to reduce pain or anxiety at the point of administering dental anesthesia in pediatric patients. The variation of heart rate and skin conductivity between the moments prior to and during anesthesia was measured, which also evaluated patient behavior and the degree of pain experienced at the point of administering anesthesia using the FLACC conformity or pain scale.

## 2. Patients and Methods

### 2.1. Patients

A controlled randomized clinical trial was conducted with 40 healthy children (16 boys and 24 girls) aged 5 to 9 years (*M* = 90 months, SD = 17.15). To be included in the sample, patients must have never received dental care and had to be seeking attention at the Pediatric Dentistry Clinic at the Autonomous University of San Luis Potosí for the first time and their dental treatment had to include a local anesthetic. The study was approved by the School of Stomatology's Research Ethics Committee at the Autonomous University of San Luis Potosí (code CEI-FE-001-016). The children's parents gave their informed consent, while the patients themselves also gave their consent.

### 2.2. Measurements

Two evaluators were trained in the correct scoring of the FLACC (Face, Legs, Activity, Cry, Consolability [16]) scale and underwent an interrater reliability evaluation. A video recording of each patient was used to score the FLACC scale during the anesthetic procedure. The evaluators were blind to the patients' group membership.

Heart rate and skin conductance data were collected with the Nexus 10 and the Biotrace software version V2015B. The blood volume sensor was placed on the index finger of the child's left hand, while the Ag-AgCL electrodes were placed on the ring and middle fingers of the same hand. For each of the 40 patients two time periods were selected. The first two minutes (baseline) were compared to the timeframe when the anesthesia was administered (minutes 12 to 14).

### 2.3. Procedure

Patients that met the inclusion criteria were randomly assigned to the experimental or control group (20 children in each). Standard conventional behavior management techniques were used with both groups, to help patients remain calm, receptive, comfortable, and relaxed.

Once the children were seated on the dental unit, the sensor and electrode functions were explained along with the sensations they could cause, before they were placed. Both groups were asked to wear headphones. The experimental group was listed to a classic directive hypnosis intervention, while the control patients were told to use headphones to block out the dental drill's noise. No sound was transmitted to the control group. The real purpose for having the control patients wear the headphones was to maintain the FLACC evaluators blind to the group membership.

The children in the experimental group were asked to listen to a recording that would teach them how their thoughts and breathing could help them feel more comfortable and relaxed. The hypnosis intervention included a standard 3-minute progressive muscle relaxation induction followed by a 5-minute deepening procedure aimed at increasing the patients' focus, absorption, and concentration. In the intervention phase the hypnotic suggestions were aimed at modifying their perception of pain. Patients were asked to visualize a safe and special garden with a fountain in the middle. They were told the fountain water would make their mouth numb and relaxed, so they would feel completely comfortable and relaxed while the dentist “made their tooth feel better.” They were then asked to raise their right arm when their mouth had gone completely numb. This ideomotor signal allowed the dentist to proceed with the anesthetic injection. Once the audio recording finished, the dentist verified that the child was completely alert and continued cooperating with the dental procedure.

### 2.4. Statistical Analysis

Statistical analyses were performed with the computing language R version 3.0.3 at a 95% confidence level. FLACC interrater reliability was assessed with both Lin's concordance correlation coefficient and Bland and Altman's concordance limits. Central and dispersion measurements were reported, with Student *t*-tests used for independent samples and multiple regression analysis. The sample size was calculated by Cohen sampling method with the possibility of type 1 error calculated at 95% and of type 2 error calculated at 80% the quantitative variability of the control or the study group estimated in 2.63; this is the minimum value between the differences we want to detect, in accordance with the results of El-Sharkawi et al. [17]; this difference was estimated at 3.59 on FLACC score, between basal measure and the application of local anesthesia by infiltration. Therefore, we took a difference of 3 measure units for our clinical significant delta which gave us a *N* = 13 patients for each group.

We made a calculation for the FLACC score (pain/anxiety, heart rate, and skin conductance), considering all of them continuous variables, and with the intention of avoiding the overparametrization, we required at least 10–20 repetitions for each degree of freedom; our analysis was based on 3 variables with 1 degree of freedom, and that is the reason why 30–60 repetitions are required.

The results are expressed as mean ± SD. The assumption of a normal distribution was evaluated with Shapiro-Wilk's test. A parametric analysis was undertaken between the measurements taken during the basal measurement and the infiltration time. The correlation among the FLACC scale, heart rate, and skin conductance was evaluated using the linear regression model, with a value of *p* ≤ 0.05 considered as statistically significant.

## 3. Results

The interrater reliability for the FLACC scale scoring in both observers yielded a Lin concordance correlation coefficient of 0.92 (CI 95%: 0.81–0.96) and a Bland and Altman concordance with a repeatability coefficient of 2.

The increase in heart rate variability (HRV) between the baseline measurement (HRV1) and the anesthesia administration (HRV2) was marginally significantly larger for the control group. However, no statistically significant differences were found for pain as measured by the FLACC scale or skin conductance (SC). See Table 1.

**Figure 1:**
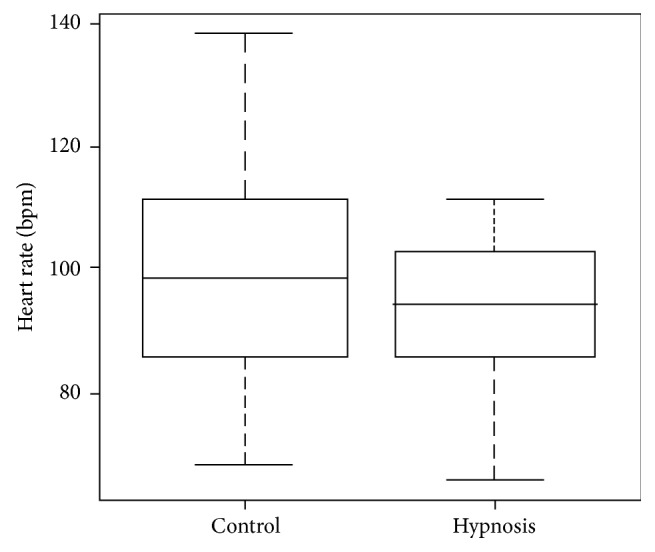
Comparison of the means of the heart rate of the groups to the anesthetic moment.

In terms of the heart rate averages at the point of administering anesthesia between the hypnosis group and the control group, the difference comprised an average obtained for the control group of 98.25 beats per minute and 94.07 beats per minute in the experimental group (Figure 1). On estimating the difference between the averages, it was determined that there was a difference of 5 beats per minute between the basal point and the point of administering anesthesia in the control group, while no difference was detected for the hypnosis group (*p* = 0.05).

Various multiple regression models were undertaken. The model shown in Table 2 presents the difference in the basal heart rate and the point of administering infiltration anesthesia to explain this variation, which included age, gender, and whether or not hypnosis was applied. This model determined that hypnotherapy is the variable that modifies the difference between heart rates. See Table 2.

## 4. Discussion

The aim of this research was to determine the dental anxiety and pain related to local infiltration anesthesia in children, in order to ascertain whether hypnosis combined with conventional behavior management techniques reduces anxiety and pain at the point of administering local anesthetic.

A less pronounced variation in heart rate was found in this study between the experimental group and the control group, which suggests that hypnosis, combined with conventional behavior management techniques, is a tool more able to help children to relax than conventional behavior management techniques alone. This tool is beneficial for the operator, as it enables optimal and more efficient clinical work, providing greater comfort and avoiding disruptive behaviors. Similar results have been obtained, presenting a reduction in the heart rate of the group submitted to hypnosis compared to the group in which it was not applied, also confirming that there is no relationship between the difference in the variation in the heart rate, age, and gender of the subjects hypnotized [8]. While only the heart rate was statistically significant in this study, clinically, a more relaxed and cooperative attitude could be seen in the subjects submitted to hypnosis.

One study measured anxiety at the point of inclusion in the research, the initial appointment, and on being seated in the dentist's chair, using a modification of the Yale scale for preoperative anxiety (mYPAS). Subsequent to the anesthesia, a visual analogue scale (VAS) and a modified objective pain score (mOPS) were used to evaluate the pain experienced. The median mYPAS and mOPS scores were significantly lower in the experimental group than in the control group, where, significantly, the hypnosis group experienced either light pain or no pain at all, suggesting that hypnosis could be effective in the reduction of anxiety and pain in children receiving dental anesthesia [16]. In contrast with this research, the abovementioned study did not use hypnosis as a complement to another therapy or a conventional behavior management technique and evaluated pain both prior to and after anesthesia. Thus, improved results could be obtained with the use of the FLACC scale alongside other scales, such as those used in the study for the evaluation of pain before, during, and after the administration of infiltration anesthesia. Furthermore, complementing the evaluation of pain and anxiety with objective methods such as those used in this study could perhaps obtain statistically significant data for the reduction of pain and anxiety during dental anesthesia.

It has been reported that the results for the use of hypnosis during local infiltration anesthesia did not present a statistically significant difference in the saturation of oxygen in terms of age, gender, ethnicity, or dental treatment undertaken. However, statistically significant differences were reported for heart rate and behavior attributable to hypnosis in terms of age, but not gender, ethnicity, or the dental treatment undertaken [8]. While statistically significant differences were found in the difference found for the heart rates, which were attributable to the hypnosis, it can be estimated that only 10% of the difference in the heart rate variation at the basal point and at the point of administering anesthesia was due to the hypnosis.

The scientific evidence regarding the use of hypnosis as a complement to another therapy for reducing anxiety during dental treatments is limited. Thus, further research is required to objectively support the reduction of anxiety during dental treatment through the use of hypnosis as a complement to other anxiety reduction techniques [15]. Similarly, as there is no evidence that hypnosis, on its own, is capable of producing an anesthetic effect for dental procedures, it should always be combined with local anesthetic techniques or as a complement to sedation or general anesthetic techniques [15].

With regard to the variations in skin conductance between the basal point and at the point of administering anesthesia, no statistically significant differences were found in this research. Furthermore, as there are few studies that support its use in dentistry, the measurement of skin conductance was not considered as an objective tool in this study. For this reason, further research is proposed into its use for the evaluation of anxiety in children undergoing dental treatment, with a larger sample complemented with other objective and/or subjective tools for its evaluation.

Furthermore, Biofeedback Nexus 10 was a precise and useful tool for monitoring heart rate and skin conductance, physiological parameters which, together with other measurements of monitorable autonomic parameters, can be considered stress indicators in situations that generate anxiety [8, 14], as was the case at the point of administering anesthesia in this research.

One of the points that should be noted from the results of this research is that it included interobserver concordance on the FLACC scale, obtaining results that enabled a reliable final evaluator trained with the evaluation instrument and which was, furthermore, blinded at all times to the group to which the subjects evaluated belonged, thus avoiding bias. Although no statistically significant differences were found between both groups studied, at the point of administering anesthesia, it would be significant to complement the use of this scale with instruments validated for the evaluation of pain or anxiety in children undergoing dental treatment.

In conclusion, evaluation using the FLACC conformity or pain scale did not present significant differences at the point of administering infiltration anesthesia for either of the two groups in this study. Similarly, the type of pain experienced by both groups was slight, with no significant differences recorded in the variation of skin conductance. However, the use of hypnosis combined with conventional pain management techniques did not show variations in terms of heart rate between the basal point and the point at which anesthesia was administered, which could show an improvement in the control of anxiety and pain in children receiving dental anesthesia.

## Figures and Tables

**Table 1:** Differences of the FLACC scale (at the time of anesthetic infiltration) and heart rate and skin conductance between groups (before and during anesthetic infiltration).

	FLACC	SC	HR, bpm	SC1	SC2	HR1, bpm	HR2, bpm
Control	2.10	−2.666	−5.767	6.0880	8.746	94.16	99.3
Hypnosis	2.65	−3.250	−1.254	7.363	10.613	92.31	93.57
*p*	0.5	0.4	0.05	0.4	0.3	0.7	0.2

Data are presented as mean. A paired Student's *t*-test was used for comparisons.

bpm = beats per minute.

**Table 2:** This model determines that hypnosis therapy is the variable that modifies the difference between heart rates. Dif HR ~ hypnosis + age + gender.

Variable	Standard error	Value of *t*	Value of *p*
Hypnosis	2.2245	2.39	0.022^*∗*^
Age	0.0656	1.12	0.272
Gender	2.3102	1.25	0.218

^*∗*^Statistically significant at *p* ≤ 0.05.

*R* ^2^ = 0.178,  *R*^2^ adjusted = 0.109, and *p* = 0.0676.
